# Novel Presentation of Aphasia and Tremor in Immune-Mediated Necrotizing Myopathy

**DOI:** 10.7759/cureus.16159

**Published:** 2021-07-04

**Authors:** Gabriella Schmuter, Julian A Paniagua-Morales

**Affiliations:** 1 Department of Internal Medicine, St. Barnabas Hospital Health System, Bronx, USA

**Keywords:** myopathy, immune-mediated necrotizing myopathy, essential tremor (et), motor aphasia, creatine kinase, necrotizing myositis

## Abstract

The authors present a unique case of immune-mediated necrotizing myopathy in a 60-year-old female who presented with aphasia, tremor, and progressive muscular weakness. To the best of our knowledge, the presentation of aphasia and tremor has not been previously reported in immune-mediated necrotizing myopathy. This rare presentation may mimic a variety of other central nervous system and myopathic disorders. Delays in prompt diagnosis and treatment of immune-mediated necrotizing myopathy can be detrimental to the physical and mental well-being of the patient and additionally leads to inefficient use of healthcare resources. Therefore, it is beneficial for clinicians to be aware of this symptomatic presentation in order to include immune-mediated necrotizing myopathy as a key differential diagnosis. Awareness of aphasia and tremor as potential symptoms of immune-mediated necrotizing myopathy will improve the quality of patient care.

## Introduction

Inflammatory myopathies include dermatomyositis, polymyositis, immune-mediated necrotizing myositis (IMNM), and inclusion-body myositis, each with incompletely defined pathophysiologic mechanisms [1,2]. Classification schemes have been proposed for stratifying each disorder according to findings on physical examination, imaging, and antibody testing [3,4]. Diagnostic techniques for proper identification of each myopathy include laboratory-based antibody testing, imaging, needle electromyogram, and muscle biopsy. MRI findings have been shown to increase the diagnostic accuracy of the muscle biopsy [2].

Various etiologies leading to the onset of IMNM include viral infections, malignancies, genetic predisposition, connective tissue disorders, medications (in particular, the use of statins) [5,6], or idiopathic presentation. Given the close association of IMNM and other inflammatory myopathies with malignancy, cancer screening is warranted in affected patients [2].

Although the pathophysiology of IMNM remains incompletely elucidated, the necrosis of fibers is thought to be commonly mediated by autoantibodies such as anti-HMGCR (anti-3-hydroxy-3-methylglutaryl-coenzyme A reductase) or anti-signal recognition particle (anti-SRP) [2,6]. IMNM is commonly associated with statin use, which pharmacologically targets HMGCR. In culture, anti-SRP antibodies have been found to demonstrate antibody-dependent complement-mediated cell lysis [2]. Levels of HMGCR or SRP antibodies have been found to correlate with alterations in creatine kinase levels. Generally speaking, IMNM is marked by profound upregulation of MHC Class I with rare lymphocytic infiltration of fibers [1]. Approximately 30-40% of patients with IMNM are seronegative [2,6]. Yet, resolution of symptoms with the introduction of immunotherapy support suggests an autoimmune etiology leading to necrotic muscle fibers in seronegative patients. Nevertheless, further investigation is needed to clarify the pathophysiologic mechanisms that mediate IMNM.

The clinical presentation of IMNM can be widely variable, commonly including distal weakness and dysphagia [7]. The classically described symptom of myopathy involves proximal muscle weakness, which may be revealed through a patient’s inability to rise from a seated position. One of the most distressing and potentially lethal concerns involves respiratory failure secondary to respiratory muscle exhaustion [1,8]. It is for this reason that prompt diagnosis and treatment of IMNM are paramount for optimal patient care and proper utilization of medical resources.

To the best of our knowledge, the symptoms of aphasia and tremor have not been previously reported in current medical literature in cases of IMNM. There are a variety of etiologies that have been previously described to present with tremor [9] or aphasia [10] that is beyond the scope of this article. Here, we present a 60-year-old woman who presented with aphasia, tremor, and proximal muscle weakness who was properly diagnosed and treated for IMNM.

## Case presentation

A 60-year-old woman presented to the emergency department at St. Barnabas Hospital Health System with chief complaints of progressive muscle weakness, tremors, and aphasia for two weeks. The patient described an inability to walk and perform daily tasks. The aphasia was classified as expressive and the tremor was most similar to essential tremor. She additionally endorsed weight loss of 20 pounds over a period of one month. Medical history was positive only for hypertension and breast cancer that was adequately treated with bilateral mastectomy 12 years ago. The patient denied any history of trauma. The patient has regularly followed up with an outpatient primary care physician to regularly obtain vaccinations and cancer screening. The patient was admitted to the Department of Internal Medicine for a comprehensive workup. 

The patient promptly obtained a computed tomography (CT) and magnetic resonance imaging (MRI) of the brain, which were found to be within normal limits. Subsequently, a full neurological examination was conducted. Examination revealed symmetrical muscle weakness in a proximal to distal gradient and a bilateral hand tremor that worsened with movement, suggesting an essential tremor. The patient demonstrated clear comprehension of speech and difficulty with word-finding and broken speech, suggesting expressive aphasia. The sensation was intact throughout, and all deep tendon reflexes were preserved. Mental status, motor function, balance, and cranial nerve testing were all intact.

Initial laboratory studies revealed elevated erythrocyte sedimentation rate, C-reactive protein, and creatine kinase. Other metabolic derangements included elevated thyroid-stimulating hormone in the presence of normal free T4, transaminitis, hypokalemia, and hypophosphatemia. Leukocytosis was notably absent. An extensive panel for antibodies of all major autoimmune diseases, including systemic lupus erythematosus, dermatomyositis, polymyositis, and immune-mediated necrotizing myopathy, were negative. Lumbar puncture was clear and absent of any oligoclonal banding.

**Figure 1:**
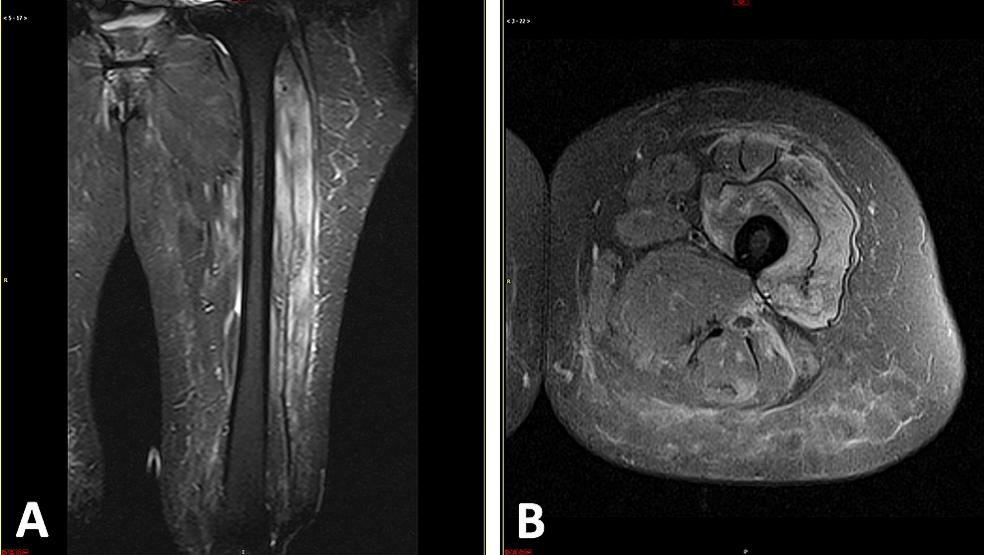
Coronal and cross-sectional MRI of the thigh (A) MRI demonstrates prominent signal hyperintensity of the left vastus lateralis and vastus intermedius muscles, with milder signal hyperintensity at the rectus femoris muscle, vastus medialis, and hamstring muscles. (B) Fluid signal intensity is visualized lateral to the biceps femoris muscle. Hyperintense inflammatory changes are visualized throughout the subcutaneous fat.

Given the negative workup at this point, it was decided to directly image the regions of proximal muscle weakness. Subsequent MRI of the femur was noted to demonstrate diffuse myositis, associated cellulitis, and mild fasciitis lateral to the biceps femoris muscle (Figure 1A and 1B). The constellation of proximal muscle weakness, elevated creatine kinase, and the MRI findings were highly suggestive of an acquired inflammatory myopathy.

**Figure 2:**
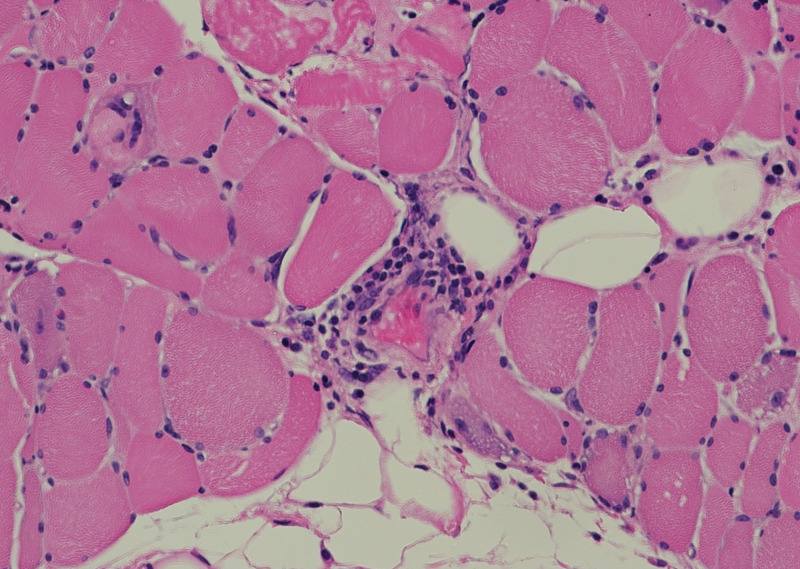
Muscle biopsy of thigh Hematoxylin and eosin staining (magnification ×20) of a left quadriceps muscle biopsy specimen demonstrates mild to moderate variation in myofiber diameter that is present with mild perivascular infiltrates of chronic inflammatory cells in the epimysium and endomysium. Enlarged reactive nuclei are present, associated with basophilic myofibers.

Neuropathology of this patient’s muscle biopsy reported diffuse chronic necrotizing process with regenerating muscle fibers, few scattered lymphocytes with no other inflammatory cells, suggestive of IMNM (Figure 2). 

Our patient was started on intravenous immunoglobulin of 2 g/kg divided over five days and a course of intravenous methylprednisolone [11,12]. Our patient improved symptomatically as her creatine kinase began to trend downward from 1405 upon admission to 260 upon discharge. She was discharged with oral prednisone prior to initiation of immunosuppressant agents with an outpatient rheumatologist.

## Discussion

Initial differential diagnoses included a cerebrovascular accident, multiple sclerosis, head trauma, brain tumor, infection, myasthenia gravis, or a hereditary or acquired myopathy. Although the chronicity of the patient’s symptoms did not suggest an acute cerebrovascular accident or transient ischemic attack, it was essential to rule out this diagnosis promptly and to investigate if her symptoms were a consequential constellation of a past stroke. Her unremarkable neurological imaging lessened the likelihood of a cerebrovascular accident, trauma, multiple sclerosis, or a brain tumor as the cause of her symptoms. In addition, the patient’s denied any history of trauma. The subacute nature of the patient’s presentation made a hereditary myopathy less likely. Negative autoantibody testing and lack of improvement of symptoms with rest lessened the likelihood of myasthenia gravis as her diagnosis. The patient’s regular visits with a primary care physician lessened the likelihood of undiagnosed malignancy as the cause of her presentation. Although the patient had a remote history of breast cancer, it was adequately treated 12 years ago and was thus less likely to be the etiology of her presentation. The patient did not possess any classical signs of multiple sclerosis such as dissemination of neurologic symptoms in time-and-space, alterations in vision, oligoclonal banding on lumbar puncture, or lesions on neurological imaging. Ultimately, our patient’s presentation of proximal muscle weakness was highly suggestive of a myopathy, which prompted our subsequent workup for this diagnosis.

The precise mechanism of injury causing IMNM remains incompletely elucidated. Standard workup warrants extensive laboratory-based antibody testing, imaging, and muscle biopsy [2], which was conducted for our patient. However, laboratory testing was not particularly specific for any single etiology in this case. The most helpful diagnostic aspect was imaging, as the MRI was highly suggestive of IMNM. With this finding, it was clinically appropriate to proceed with a muscle biopsy to solidify the suspicion of the diagnosis.

The current mainstay of treatment for IMNM involves corticosteroids and at least two immunosuppressant medications [11,12]. Our patient displayed marked symptomatic improvement given prompt initiation of the standard treatment protocol. Other treatment options, such as rituximab [13], are available for those with IMNM who are resistant to treatment with steroids and multiple immunotherapies. In addition to recovering proximal muscle strength with physical therapy, the patient’s aphasia and tremor resolved completely within a few days of initiating treatment. In this context, it is suggested that the patient's aphasia and tremor was directly related to her acute condition rather than coincidentally overlapping with the presentation.

Tremor is generally considered to be a frequent symptom in involuntary movement disorders with its classification based on the situation in which the action is present. Each type of tremor is associated with degeneration of a particular neuronal circuit [14]. Aphasia is classically seen in post-stroke patients as a result of disruption of brain networks associated with production or comprehension of language [15]. Aphasia has been associated with negatively impacting the quality-of-life of affected individuals [16,17]. In addition to recovering proximal muscle strength with physical therapy, the patient’s aphasia and tremor resolved completely within a few days of initiating treatment. In this context, it is suggested that the patient's aphasia and tremor was directly related to an autoimmune etiology. Given the fact that her symptoms of aphasia and tremor emerged and resolved within the same chronicity of her IMNM episode, it is highly suggested that these symptoms originated from IMNM rather than an alternative etiology.

Nevertheless, the presence of aphasia and tremor in our patient was unexpected. It is possible that our patient may have presented with an autoimmune disruption of neuronal circuitry leading to such symptoms. The patient may have experienced autoimmune damage to the frontal region of the language-dominant side of her brain, particularly around Broca’s area leading to broken speech. The lack of remarkable findings on neurological imaging challenges our ability to pinpoint which regions of the brain may have been impacted during this episode. There have been previous reports of isolated primary progressive aphasia resolving with immunosuppressant treatment, revealing that an autoimmune etiology may lead to such symptom [18]. Further investigation of the mechanism of tremor and aphasia is warranted within the realm of inflammatory myopathies. Awareness of this potential presentation in the clinical setting is important for avoidance of complicated sequelae from late or missed diagnosis of IMNM.

## Conclusions

Aphasia, tremor, and proximal muscle weakness were observed in a 60-year-old female with the diagnosis of IMNM. This constellation of symptoms involving aphasia and tremor has not been previously documented in the medical literature. Awareness of this potential presentation is essential for prompt diagnosis and treatment in the clinical setting, particularly given the potentially detrimental and lethal course that is possible in a myopathic derangement. With the recognition of these unique symptoms, timely diagnosis and treatment of IMNM will improve the overall quality of patient care and will efficiently use medical resources.
